# MEFV Gene Variant Alleles in Normal Population of Northwest of Iran, Which Is Near to Mediterranean Sea

**DOI:** 10.1155/2019/6418759

**Published:** 2019-08-18

**Authors:** Farhad Salehzadeh, Afshan Sharghi, Atena Motayayagheni, Saied Hosseini Asl, Mahsa Mottaghi, Sepehr Sarkhanloo

**Affiliations:** ^1^Pediatric Rheumatology, Pediatric Department, Bouali Children's Hospital, Ardabil University of Medical Sciences (ARUMS), Ardabil, Iran; ^2^Ardabil University of Medical Sciences (ARUMS), Ardabil, Iran; ^3^Pediatric Department, Bouali Children's Hospital, Ardabil University of Medical Sciences (ARUMS), Ardabil, Iran; ^4^Molecular-Genetic Laboratory, Imam Khomeini Hospital, Ardabil University of Medical Sciences, Ardabil, Iran

## Abstract

**Background and Objective:**

* MEFV *gene codes the pyrine protein that has major role in FMF as an autoinflammatory disorder. FMF is more often seen in the people of the Mediterranean area. Considering the significant role of* MEFV *gene in many rheumatologic diseases and even nonrheumatologic disorders, it is necessary to identify different variations of these mutations in the healthy and normal population of this area.

**Methods:**

224 healthy (unaffected or control) people based on the Cochran formula entered this study. The blood samples were screened for the 12 common* MEFV *gene variants polymorphisms according to manufacturer's instructions (FMF Strip Assay, Vienna lab, Vienna, Austria). They filled a questionnaire containing required information. All healthy control cases initially were evaluated for FMF symptoms and signs in themselves and their first-degree relatives based on clinical criteria. All data were analyzed by simple statistical method.

**Results:**

Among 224 healthy control cases, 113 (50.4%) were male and 111 (49.6%) female. There were* MEFV *variants alleles in 57 patients (25%): 28 were male (49.1%) and 29 female (50.9%). The most frequent variants were E148Q (18.3%), followed by P369S (3.1%), V726A (2.2%), A744S (1.3%), and F479L, M694V, and R761H (0.8%), and eventually K695R (0.4%), respectively. Some variants such as M694I, M680I (G/C), M680I (G/A), and I692del were not seen in these samples. There were compound heterozygote variations of E148Q/P369S, E148Q/V726A, E148Q/P369S, and P369S/F479L in normal population without any findings in favor of FMF.

**Conclusion:**

Twenty-five percent of the normal populations of the northwest of Iran are carrying* MEFV *gene variants, and the most common mutation is E148Q (18.3%). The presence of M694I, M680I (G/C), M680I (G/A), I692del mutations in the normal population can be interpreted cautiously, while particular compound heterozygote mutations can be considered as normal variants.

## 1. Introduction

FMF disease is a self-inflammatory autosomal recessive genetic disorder inherited through the* MEFV *gene. FMF is more often seen in the people of the Mediterranean coast, Turkish breeds, Jews, Arabs, and Armenians. The prevalence of this disease in high-risk populations is about 1-3 per 1,000, and it is rare in other ethnic groups. The gene responsible for the disease is the* MEFV *gene [1–3].

The location of this gene is on the chromosome 16 (P13.3).* MEFV *gene encodes a protein, called Pyrin, which plays a role in reducing inflammation. The inflammatory regulation does not occur correctly in mutated condition. More than 342 sequence variants have already been identified on* MEFV *gene [4–7].

FMF is characterized by recurrent fever and inflammation of the serous membranes, which causes abdominal pain, joint inflammation, and chest pain. Some other diseases that are characterized by inflammation of the blood vessels (vasculitis), such as Henoch-Schönlein purpura and polyarteritis nodosa, are more common in FMF patient. The most severe complication in untreated patient is amyloidosis [8].

Symptoms can be manifested in the first decade of life; near 83% of the patients show the first signs of the disease during childhood and adolescences [9, 10]. Diagnosis of FMF is based on the Tel-Hashomer clinical criteria and confirmation by* MEFV *gene mutations evaluation. Among common polymorphisms M694V is one of the most frequent variants and causes the most severe clinical features [11]. FMF is considered in the differential diagnosis of different autoinflammatory syndromes and different studies discussed about the possible role of the MEFV gene in PFAPA patients [12].

Considering the importance of FMF and its significant prevalence in the northwest of Iran, near Mediterranean region [1], and the significant role of* MEFV *gene in many rheumatologic diseases such as Behcet's disease, PAN, HSP, and even nonrheumatologic disorders [3], it is necessary to identify different variations of this mutations in the unaffected and normal population of this area, which is near to Mediterranean Sea.

## 2. Methods

This descriptive cross-sectional study was conducted on peoples over 40 years old who were referred to the tertiary central Hospital during two years for cardiovascular evaluations.

Including criteria were as follows:

- People over 40 years old.
- Absence of FMF and periodic fever symptoms in cases and their first-degree relatives based on clinical and genetic criteria.
- Lack of rheumatologic diseases such as RA, Behcet's disease, and vasculitis in patients.

The number of samples is determined based on the Cochran formula, as 224 subjects. Blood samples were screened for the 12 common pathogenic variants (E148Q, P369S, F479L, I692del, M680I(G/C), M680I(G/A), M694V, M694I, K695R, V726A, A 744S, and R 761H) according to manufacturer's instructions (FMF Strip Assay, Vienna lab, Vienna, Austria).

Patients filled out a questionnaire containing complete information of their past medical history including rheumatologic disorder. All patients initially were examined and evaluated for the FMF symptoms and presentations.

All data were analyzed by simple statistical method.

The study was approved by the faculty of Medicine Ethics Committee and informed consent was obtained from all the participants.

## 3. Results

From 224 samples 113 (50.4%) cases were male and 111 (49.6%) female.* MEFV *gene variants were detected in 57 cases (25%); among them 28 were male (49.1%) and 29 female (50.9%). There was not any correlation between sex and* MEFV *mutations.

The most frequent variant was E148Q in 41 cases (18.3%), followed by P369S with 7 cases (3.1%), V726A with 5 cases (2.2%), A744S with 3 cases (1.3%), and F479L, M694V, and R761H each of them with 2 cases (0.8%) and eventually only 1 case (0.4%) of the K695R mutation. M694I, M680I (G/C), M680I (G/A), and I692del mutations were not seen in these unaffected people (Table 1).

There were also some compound heterozygote variants such as E148Q/P369S, E148Q/V726A, E148Q/P369S, and P369S/F479L in normal population in this area. There was significantly presence of P369S mutation in compound asymptomatic heterozygote variants.

## 4. Discussion

This study showed 25% of* MEFV *gene variants in the normal population of northwest of Iran, which is near to the Mediterranean Sea and possesses high prevalence of FMF disease.

In a study by Coşkun et al. [13], 220 patients with FMF were compared with a group of 228 healthy people as a control group. Five mutations of M694V, M694I, M680I, V726A, R761H, and E148Q were determined. The E148Q mutation similar to our study was found to be significantly higher in the control group (10.5%).

Four cases had compound heterozygote mutations (E148Q/P369S, E148Q/V726A, E148Q/P369S, and P369S/F479L); none of them had symptoms of FMF and serositis or other rheumatologic diseases. This emphasized the numerous studies that occurrence of FMF is based on genetic factors, but different environmental factors could influence it [14].

In a study by Bonyadi et al., five MEFV mutations (M694V, V726A, M680I, M694I, and E148Q) were examined in 200 healthy people. They showed 25% mutation in normal Azeri Turkish population. The most common mutation was E148Q with 11.5% and subsequently V726A with 1.75 % [15].

Our study was carried out with examining 12 common mutations and showed the same results with a higher percentage, E148Q mutation with 18.3%, and V726A mutation with 2.2%.

In a study by Ebadi et al., twelve common MEFV gene mutations were examined in 390 FMF patients from all area of Iran. 234 patients (60%) had at least one mutation, and 156 patients (40%) were without any common mutations. The most common variants were M694V (13.6%), followed by E148Q (10.4%), M694I (6.5%), V726A (4.1%), and M680I (3.8%), respectively [16].

In the study of Salehzadeh et al. [1], twelve common MEFV gene mutations in 216 patients with FMF in the northwest of Iran were examined; the most common alleles were M694V (23.6%), V726A (11.1%), and E148Q (9.9%). This study in the normal population of the same region represents an entirely different genotype of the patients and normal population (Table 2).

It can be concluded that if E148Q mutation is seen in an individual without FMF symptoms, it should be considered as a normal variant rather than being a pathologic mutation associated with FMF disease.

In this area the presence of M680I (G/C), M680I (G/A), I692del, and M694I variants in asymptomatic people should be interpreted with caution and considered in favor of disease, particularly in suspected cases; moreover, the E148Q/P369S, E148Q/V726A, E148Q/P369S, and P369S/F479L compound heterozygote variants could be interpreted as normal variants, even with two types of compound heterozygote variants.

## 5. Conclusion

Twenty-five percent of the normal populations of the northwest of Iran have* MEFV *gene variants alleles and the most common variant is E148Q (18.3%). It seems M694I, M680I (G/C), M680I (G/A), and I692del mutations are not a normal population variants.

## Figures and Tables

**Table 1:** Frequency of variant alleles.

Variant Alleles	No (%)
E148Q	41 (18.3)
P369S	7 (3.1)
V726A	5 (2.2)
A744S	3 (1.3)
F476L	2 (0.8)
M694V	2 (0.8)
R761H	2 (0.8)
K695R	1 (0.4)

**Table 2:** Comparison of patients and normal populations.

Variant Alleles	FMF	Normal
	patients	populations
M694V	23.6%	0.8%
V726A	11.1%	2.2%
E148Q	9.9%	18.3%
M680I	9%	-
R761H	3.4%	0.8
M694I	3%	-

## Data Availability

The data used to support the findings of this study are available from the corresponding author upon request.

## Abbreviations

FMF: Familial Mediterranean fever
PAN: Polyarteritis nodosa
HSP: Henoch-Schönlein purpura
MEFV gene: Mediterranean fever gene
PFAPA: Periodic fever with aphthous stomatitis, pharyngitis, and cervical adenitis.
